# Melatonin as a Radio-Sensitizer in Cancer

**DOI:** 10.3390/biomedicines8080247

**Published:** 2020-07-27

**Authors:** Carolina Alonso-González, Alicia González, Javier Menéndez-Menéndez, Carlos Martínez-Campa, Samuel Cos

**Affiliations:** Department of Physiology and Pharmacology, School of Medicine, University of Cantabria and Instituto de Investigación Sanitaria Valdecilla (IDIVAL), 39011 Santander, Spain; carolina.alonso@unican.es (C.A.-G.); javier.menendezm@alumnos.unican.es (J.M.-M.); coss@unican.es (S.C.)

**Keywords:** melatonin, radiotherapy, radiosensitization, cancer cells

## Abstract

Radiotherapy is one of the treatments of choice in many types of cancer. Adjuvant treatments to radiotherapy try, on one hand, to enhance the response of tumor cells to radiation and, on the other hand, to reduce the side effects to normal cells. Radiosensitizers are agents that increase the effect of radiation in tumor cells by trying not to increase side effects in normal tissues. Melatonin is a hormone produced mainly by the pineal gland which has an important role in the regulation of cancer growth, especially in hormone-dependent mammary tumors. Different studies have showed that melatonin administered with radiotherapy is able to enhance its therapeutic effects and can protect normal cells against side effects of this treatment. Several mechanisms are involved in the radiosensitization induced by melatonin: increase of reactive oxygen species production, modulation of proteins involved in estrogen biosynthesis, impairment of tumor cells to DNA repair, modulation of angiogenesis, abolition of inflammation, induction of apoptosis, stimulation of preadipocytes differentiation and modulation of metabolism. At this moment, there are very few clinical trials that study the therapeutic usefulness to associate melatonin and radiotherapy in humans. All findings point to melatonin as an effective adjuvant molecule to radiotherapy in cancer treatment.

## 1. Introduction

Radiotherapy is widely used for curative or palliative patient malignancies in many types of cancer [1,2,3]. It has been assessed that nearly 80% of cancer patients received radiation therapy alone or in combination with chemotherapy or hormonal therapy [4]. Besides its well-known properties, ionizing radiation has also been linked to numerous deleterious effects, produced by direct or indirect mechanisms. Thus, cell signaling is directly affected by the production of highly reactive oxygen species (ROS) involved in oxidative damage which may result in apoptosis, cell growth inhibition or EMT (epithelial-to-mesenchymal transition) transformation [5]. Despite technological advancements in radiotherapy that allow the administration of high doses of radiation to a specific and well-defined area, the irradiation of some degree of the surrounding normal tissue is still unavoidable. Adjuvant treatments to radiotherapy have two main objectives, on one hand, to enhance the response of tumor cells to radiation treatment and, on the other hand, to reduce side effects to normal tissues. For this reason, the two therapeutic strategies employed are the use of radiosensitizers targeted above all to cancer cells and radioprotectors for reducing deleterious side effects in the normal cells. Radiosensitizing agents should increase the effect of radiation in tumor cells without increasing side effects in normal tissues. Moreover, radiosensitizers have a potential role contributing to overcoming resistance to radiation, a major therapeutic problem. Hypoxia and nutrient depletion in the tumor can influence the response to treatments in such a way that cancer cells in a hypoxic microenvironment may be as much as three times more resistant to radiation damage. Thus, developing effective radiosensitizers is critical to improve the curative effects of radiotherapy. Several approaches have been recently tested: the use of inhibitors of the EGFR/mTOR/HIF-1 (epidermal growth factor receptor/mammalian target of rapamycin/hypoxia inducible factor-1) signaling pathway, such as cetuximab [6], the use of silica nanoparticles to deliver hypoxia-activated prodrugs such as tirapazamine [7], the development of nitroimidazole analogues [8], HSP90 inhibitor such as ganetespib [9] or the identification of miRNAs secreted by hypoxic cancer cells [10]. In the same way, radioprotector agents should decrease the adverse effects on normal tissues without reducing the efficacy of radiation on tumor tissue. 

In this review, we will compile the main actions of ionizing radiation, the pathways involved in the cell response to irradiation, some of best known radiosensitizers and finally we will describe some of the mechanisms through which melatonin is known to sensitize cancer cells to ionizing radiation.

## 2. Biological Effects of Radiation

The main target of ionizing radiation is DNA. An ionizing radiation may pass through cells and directly interact with DNA or ionize water molecules producing highly reactive radicals (reactive oxygen species and reactive nitrogen species) which interact with DNA and other organelles in tumor cells. This action may damage a nucleobase, cause a break in the sugar-phosphate backbone and produce a single- or a double-strand break or a DNA crosslink. For cell survival, the damage of a base or a single-strand break is not relevant because cells have mechanisms, like base excision repair mechanism, to fix the break [11]. In addition, most of the double-strand breaks induced by low linear energy transfer are also repaired by cells. However, a small proportion of the double-strand breaks induced by low linear energy transfer and most of the double-strand breaks induced by high linear energy transfer are not repaired by cells due to its great complexity, and therefore they lead to cell death, senescence, mutations or genomic instability. Cell response may be either the death of the cell or the genesis of a neoplasia. Damaged cells and adjacent lymphocytes and macrophages are involved in the activation of pathways related to inflammation, DNA repair and redox metabolism. Besides free radicals production, these processes stimulate the synthesis of many cytokines, such as IL-1, IL-4, IL-6, IL-8, IL-13, TNF-α or TGF-β that play a key role in the chronic oxidative stress and are also involved in the DNA breaks and cell death [12,13,14].

## 3. Signaling Pathways of the Cellular Radiation Response

Several cellular pathways are involved in the cellular radiation response that leads to survival or induction of either cell death or senescence of cells.

### 3.1. p53

One of the first stage in the response of cells to DNA damage is the cell cycle arrest mediated by ATM (ataxia-telangiectasia mutated), a main regulator of cell response to the double-strand break produced by ionizing radiation. The arrest of the cell cycle allows the repair of the DNA. Then, the activation of ATM phosphorylates and activates p53. Depending on cell damage and alterations of p53, the survival pathway or the cell death pathway is activated. For cell cycle arrest, p53 enhances the expression of the cyclin-dependent kinase (CDK) inhibitor p21, which arrests cell cycle progression during the G_1_ and G_2_ phases [15,16].

### 3.2. DNA Double-Strand Break Repair Pathways

DNA double-strand breaks are repaired by two pathways, non-homologous end joining and homologous recombination. The chosen mechanism depends on the phase of the cell cycle. Homologous recombination is a complex error-free pathway, which requires the proximity of the sister chromatid and because of that, only takes place in late S and the G_2_ phases of the cell cycle, before the cells enter mitosis [17,18]. However, non-homologous end joining repairs the broken ends without the requirement of a second template and is preeminent in G_1_ phase [19,20]. Some of the main elements of the non-homologous end-joining pathway are the Ku70/80 heterodimer and the catalytic subunit of DNA-dependent protein kinase (DNA-PKcs), which together make the active DNA-PK, XRCC4, ligase IV and the endonuclease artemis [20,21]. The over or under-expression of DNA-PKcs has been related with highly sensitivity or resistance to ionizing radiation [22,23,24]. The homologous recombination is initiated by the MRN(Mre11, Rad50, Nbs1), complex and CTIP (CtBP-Interacting Protein). In this process RAD51 plays a main function developing nucleoprotein filaments on single-strand DNA before the strand exchange. After the double-strand break, BRCA2, a complex that recruits RAD51 to sites of DNA damage, is activated by phosphorylation. 

When the damage in DNA after radiation is solved, cells will progress through the cell cycle but, if the repair is not adequate, cells will be directed to cell death, either by apoptosis, mitotic catastrophe or senescence, depending on the dose of radiation, the phase of the cell cycle in which the cells are, the cell type or the presence or not of oxygen [16,25].

### 3.3. Apoptosis

Irradiation induces apoptosis primarily through both the intrinsic and the extrinsic apoptotic pathways and also through the membrane stress pathway. The intrinsic apoptotic pathway is associated with mitochondria and is influenced by proteins of the BCL (B-cell lymphoma family) family, such as Bax and Bcl-2 which are respectively anti-apoptotic and apoptotic. Radiation also induces apoptosis through the extrinsic pathway mediated by the death receptors, mainly those belonging to the tumor necrosis factor receptor (TNFR) family. Intrinsic and extrinsic apoptotic pathways are mediated by the activation of p53 in the DNA damage response. The membrane stress apoptotic pathway is a DNA damage-independent apoptotic process that does not require p53 activation and is mediated by the release of ceramide of the plasma membrane [16].

### 3.4. Mitotic Catastrophe

Mitotic catastrophe is the most relevant kind of cell death induced by ionizing radiation. It is a form of cell death based on induction of mitosis before completing the S and G_2_ phase. It is triggered by a combination of deficient cell-cycle checkpoints and cellular damage and, as a consequence, cell death is induced in the next division following irradiation [16].

### 3.5. Senescence

The activation of p53 after irradiation often leads to activation of p21. This fact induces a permanent cell-cycle arrest that causes senescence. Many tumor cells respond to ionizing radiation by triggering stress-induced premature senescence. Senescent cells are metabolically active and can sometimes secrete growth factors that stimulate tumor growth. Senescence in tumor cells has been described as a way to escape from cytotoxic effects of radiation [16,26].

### 3.6. Autophagy

Autophagy is a process of lysosomal degradation of organelles through the formation of autophagosomes. In response to radiation-induced stress, autophagy has two different functions in cancer cells, on the one hand cytoprotection, whose inhibition can sensitize cancer cells to radiation; on the other hand cytotoxic function, which induces the death of cancer cells [27]. Cancer cells often utilize autophagy as a survival mechanism against different kinds of stress caused either by chemotherapy or radiotherapy. Autophagy has been proposed to be responsible for radio-resistance by eliminating ROS-produced damage [16,28].

### 3.7. EGFR (Epidermal Growth Factor Receptor) Signalling

Ionizing radiation can mimic the effects of ligand binding to EGFR and stimulates cell cycle progression and proliferation and inhibit apoptosis [16,29,30]. In addition, the irradiation of EGFR induces DNA repair by activation of non-homologous end joining and homologous recombination [16,29].

## 4. Radiosensitizers

Radiosensitizers are agents used in the treatment of cancer to augment cancer cell damage after irradiation by increasing the sensibility of tumor cells. Depending on their structure, the radiosensitizers can be classified in three groups: small-molecules, nanostructures and macromolecules.

Oxygen and small molecules with similar characteristics fix the damage, avoiding repair by reductants that contain a thiol group, such as glutathione, neutralizing free radicals which results in an increase of the efficacy of ionizing radiation. Some of the small molecules known to function like radiosensitizers are, among many others: nocotinamide, clofibrate, misonidazole, etanidazole, amifostine, capecitabine, SN30000 and AQ4N. Some Hypoxia-specific cytotoxins with high cytotoxicity towards hypoxic cells, such as tirapazamine, increase the damage induced by radiation. The efficacy of some sensitizers (HDAC4, PIeK-Akt-mTOR, RSU1069, etc) has been related with their influence in different signaling pathways of DNA repair, apoptosis, metastasis, etc. [30]. The use of modified nucleosides is another option. Incorporation of 5-substituted uracils or 5-bromo-2’-deoxyuridine into genomic DNA sensitizes cellular DNA to radiation. The reactive nucleobase radical formed as a primary product quickly stabilizes and leads to DNA damage, like cross-links or strand breaks [31,32]

A new way to expand the diversity of radiosensitizers has been the development of nanostructures. Nanomaterials are able to interact with ionizing radiation at chemical, physical and biological levels by enhancing ROS production, stimulating oxidative stress and sensitizing DNA by direct binding, resulting in the increase of the efficacy of irradiation. Metallic nanomaterials or nano delivery systems are strategies employed to increase radiosensitizer concentrations at tumor cell level [33].

Finally, a third group of radiosensitizers include macromolecules, such as microRNAs, proteins, peptides, oligonucleotides, siRNAs, which have been associated with the regulation of sensitivity to ionizing radiation through different mechanisms: complementary binding to DNA, down or up-regulation of miRNAs, interaction with key proteins, delivery of radiosensitizers, etc. [34,35,36].

## 5. Melatonin: An Antitumor Hormone

Melatonin is a natural hormone produced mainly by the pineal gland which is ubiquitously distributed and exerts an important role in the regulation of cancer growth, especially in hormone-dependent mammary tumors. It is well known from animal models that melatonin diminished the incidence and growth of breast tumors, whereas pinealectomy usually stimulates breast cancer growth. Also, in *in vitro* human breast cancer cells models, melatonin shows a wide broad of oncostatic actions inhibiting cell proliferation and invasiveness and also promoting apoptosis [37,38]. This indolamine exerts many of its actions through binding to specific G-protein membrane receptors (MT_1_, MT_2_) but also through some other mechanisms like binding to orphan receptors or interaction with intracellular molecules such as calmodulin [39]. 

**Figure 1:**
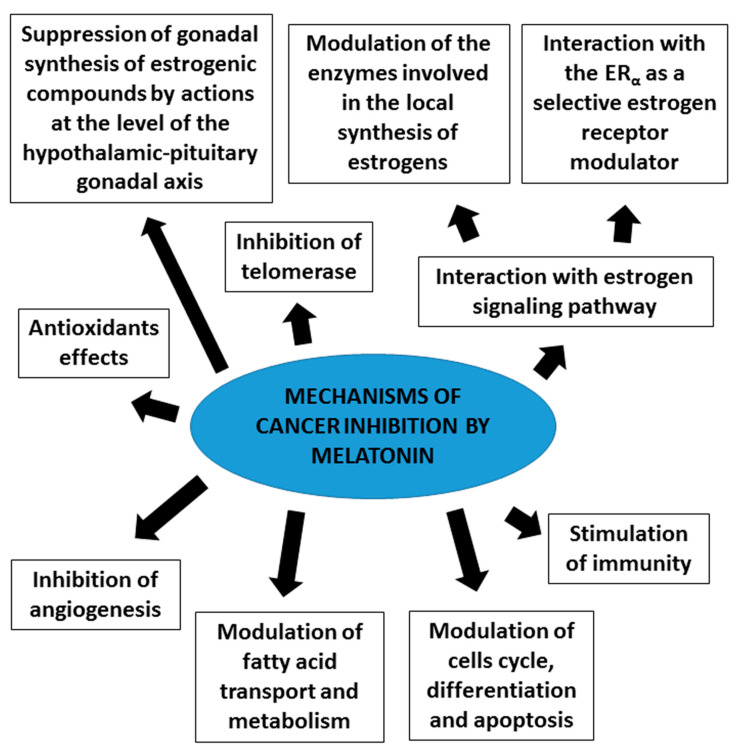
Mechanisms involved in melatonin antitumor actions.

It has been confirmed that melatonin exerts oncostatic actions through different biological mechanisms (Figure 1), such as: indirect effects of melatonin at the level of the hypothalamic-pituitary-reproductive axis, which induces a decrease of biosynthesis of some of the hormones that stimulate the proliferation of cancer cells, such as the estrogenic compounds produced by the gonads [40]; antiestrogenic actions of melatonin directly at cancer cell level [41,42]; enhancement of the anticancer immunity and hemopoiesis [43]; inhibition of telomerase in cancer cells [44,45]; reduction of angiogenesis [46,47,48]; antioxidant effects [49]; inhibition of fatty acid uptake and fat metabolic pathways [50,51] and modulation of cell cycle, differentiation and apoptosis [38].

## 6. Radiosensitizing Effects of Melatonin on Cancer: Mechanisms Involved in the Radiosensitization Induced by Melatonin

In recent years several studies have showed that melatonin administered with radiotherapy is able to enhance its therapeutic effects and can protect normal cells against side effects of this treatment. The radiosensitizing effects of melatonin have been described both in *in vivo* and *in vitro* studies and many reports have identified a variety of mechanisms that explain how melatonin potentiates the effects of radiation (Table 1).

### 6.1. Increase of Reactive Oxygen Species (ROS) Production

Free radicals and oxidative stress have been frequently related to cancer. ROS play a main role in the regulation of several functions, such as proliferation and apoptosis. Among the oncostatic actions of melatonin, one of the mechanisms described has been its antioxidant effect, which has been deeply explored since 1993, when it was first defined that melatonin is a free radical scavenger [78]. These antioxidative effects of melatonin classify into two types of actions: direct actions as a free radical scavenger, such as reactive oxygen and nitrogen species, and indirect effects through the regulation of the activity and gene transcription of antioxidant or pro-oxidant enzymes [79,80]. Melatonin at high concentrations (500 μM) potentiates the acute cytotoxic effects of radiation in head and neck squamous cell carcinoma. The improvement of the actions of radiation is partly due to enhanced mitochondrial function. Melatonin induces apoptosis in head and neck squamous cell carcinoma by generating intracellular ROS, which play a role in mitochondria-mediated apoptosis and autophagy [52]. In these kinds of tumors, melatonin combined with irradiation enhances the increase of expression of ATG12-ATG5, a complex essential for autophagosome formation, induced by radiation [52]. In addition, melatonin potentiates laser irradiation by increasing the content of generated intracellular ROS, particularly in ovarian carcinoma cells and endothelial cells adjacent to tumor cells. An increase of HSP70, associated with the generation of ROS induced by laser irradiation, was also enhanced by melatonin. HSP70 has an important function in apoptosis and in the defense of cells against stress oxidative status. Melatonin potentiates laser irradiation effects by stimulation of free-radical mediated apoptosis [53]. In human cervical cancer cells, the effects of melatonin on the radiosensitivity are dose-dependent and a concentration of 10 μM enhances the cell-killing effect of X-rays irradiation, mediated by an increase in ROS induced by melatonin, whereas 1 mM concentration neutralizes X-rays irradiation-induced cell inactivation [54].

Melatonin can either stimulate or inhibit signal transduction pathways in a receptor-dependent or receptor-independent manner. It binds with high affinity to the membrane receptors in the picomolar range. In the nanomolar range, it can interact with nuclear receptors (RZR/ROR) as well as to calmodulin. At millimolar concentrations, it can behave as an antioxidant compound [81]. Administration of melatonin 10 μM increased the cell killing effect caused by X-rays irradiation, mainly by activation of the c-Jun NH2-terminal kinase signaling, depending on the binding of the pineal hormone to its melatonin receptor on the membrane [81]. Radiosensitization by melatonin of thyroid cancer cells have been related with an increase of reactive oxygen species and an inhibition of p65 phosphorylation, a protein involved in NF-kB (nuclear factor kappa-light-chain-enhancer of activated B cells) signaling [55].

### 6.2. Modulation of Proteins Involved in Estrogen Biosynthesis

The estrogen-signaling pathway is a main target in hormone-dependent cancer treatment. Since estrogens are associated with the genesis and growth of breast cancer, one of the objectives in the malignant mammary tumors therapy has always been to neutralize the action of estrogens. Some examples of therapies with this goal are: ovariectomy, the usage of drugs that interfere with the action of the estrogens at the estrogen receptor level on the breast, or drugs that modulate the enzymes involved in the estrogen biosynthesis. On the other hand, since the pineal gland and the melatonin, its main secretion product, are involved in the inhibition of the gonadal maturation and sex hormone secretion in mammals, a relationship between melatonin levels and the genesis and growth of malignant estrogen-responsive tumors was established [82,83]. Melatonin, at tumor cell level, disrupts estrogen-dependent pathways by reducing estrogen effects through regulating both the estrogen receptor expression and its transactivation. In addition, melatonin modulates the activity and expression of the enzymes involved in the local synthesis of estrogens at human breast cancer cell level. In the normal mammary tissue, the local estrogen biosynthesis tends towards estrone production from the circulating inactive precursors androstenedione and estrone sulfates, whereas in the breast cancer tissues tends towards the production of the more potent 17β-estradiol. In malignant breast tumors, aromatase, which converts androstenedione into estrone, sulfatase, which transform the estrone sulfates to estrone, and 17β-HSD1, which converts the estrone to the more active 17β-estradiol, are often overexpressed. The expression of estrogen sulfotransferase, the enzyme that inactivates estrone and 17β-estradiol, is frequently decreased. As a consequence, the levels of 17β-estradiol in breast cancer tissues are elevated. Melatonin modifies the activity and expression of these enzymes by taking them to values similar to those observed in normal tissue. Thus, melatonin decreases the activity and expression of aromatase, sulfatase and 17β-HSD1 and increases the activity and expression of estrogen sulfotransferase, and in this way, it reduces the estrogenic effects on the mammary tissue [84,85,86].

**Figure 2:**
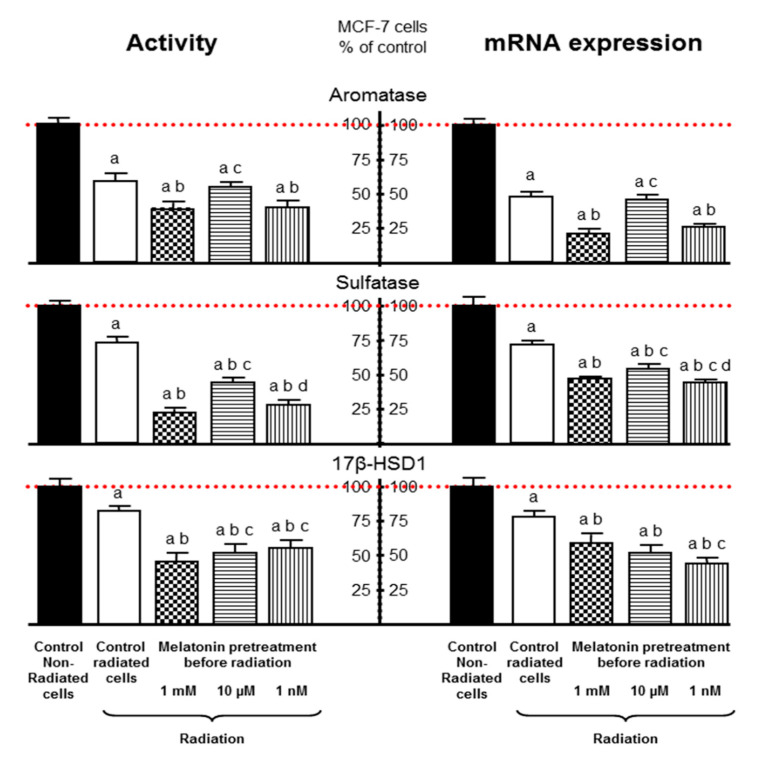
Modulation of proteins involved in estrogen biosynthesis. Melatonin pre-treatment (1 mM, 10 µM or 1 nM) enhances the inhibitory effect induced by the radiation on aromatase, sulfatase and 17β-HSD1 (17β-Hydroxysteroid dehydrogenase) activity and expression of MCF-7 cells. Data are expressed as the percentage of the control non-radiated group (mean ± SEM). (**a**), *p* < 0.001 vs. control non-radiated cells; (**b**), *p* < 0.001 vs. control radiated cells; (**c**), *p* < 0.01 vs. 1 mM melatonin; (**d**), *p* < 0.001 vs. 10 µM melatonin. Modified from Alonso-González et al. [57].

Alonso-González et al. (2016) described that melatonin treatment before ionizing radiation increases the cytotoxic and radiosensitizing effects in human breast cancer cells and that is associated with the regulation of several proteins involved in estrogen biosynthesis [56]. Radiation decreased aromatase, sulfatase and 17β-HSD1 activity and expression by itself and melatonin pre-treatment enhanced this inhibitory effect induced by the radiation (Figure 2). The down-regulation on the aromatase gene expression induced by melatonin was due to the inhibition of the two main specific aromatase promoter regions pII and pI.3 in breast cancer tissue. These authors suggest that the up-regulation of p53 could be the link between melatonin and its regulatory effect on breast cancer cells sensitivity to the ionizing radiation. p53, besides being a regulator of effectors which promote cell cycle arrest, DNA repair or apoptosis induction, is a negative modulator of aromatase in the breast [57,58].

Recently, it has also been demonstrated that melatonin sensitizes, not only cancer cells but also peritumoral cells, such as endothelial cells or breast pre-adipocytes. Melatonin at 1 mM concentration potentiated the inhibitory effect exerted by ionizing radiation in human endothelial cells on several stages of the angiogenic process, such as endothelial cell proliferation, migration or tubular network formation, as well as on the enzymes implicated in estrogen biosynthesis [59]. In co-cultures of human endothelial cells and breast cancer cells, radiation decreased aromatase mRNA expression, sulfatase activity and expression and 17β-HSD1 activity and mRNA expression, and melatonin pre-treatment significantly increased this inhibitory effect of radiation [59]. The regulation of aromatase expression was exerted through an inhibition of the aromatase promoter I.7, the most active aromatase promoter in endothelial cells.

Breast adipose fibroblasts, like endothelial cells, are a source of estrogen biosynthesis. Radiation also decreased aromatase activity and expression in breast adipose fibroblasts. Melatonin pre-treatment enhanced the down-regulation of aromatase activity and expression induced by radiation [60]. This melatonin effect was mediated through an increase in the inhibition of aromatase promoter II mRNA expression. In addition, it is known that cyclooxygenases (COX) enzymes are associated with aromatase expression through an increase of PGE_2_ (Prostaglandin E_2_), which raises the levels of intracellular cAMP, that enhances promoter II and I.3 mediated transcription, thus increasing aromatase expression. Radiation decreased COX-1 and COX-2 mRNA expression and melatonin enhanced this inhibitory effect of radiation [60].

### 6.3. Impairment of Tumor Cells to DNA Repair 

The efficacy of treatments like ionizing radiation or chemotherapeutics agents in cancer has been associated with the mechanisms of DNA repair [61,87]. DNA double-strand break (DSB) is the main cytotoxic lesion exerted by ionizing radiation that promotes cell-cycle arrest and then cell death [62]. An enhancement in DNA repair capability has been involved in therapy resistance [88]. Homologous recombination and non-homologous end-joining are the two pathways that mediate the repair of DSBs [89]. RAD51 is a protein that plays a main role in the homologous recombinant mediated repair of double-strand break and its levels are increased in cancer cells [90]. DNA-PKcs play a main role in the non-homologous DNA end joining and its overexpression in cancer cells is involved in resistance to ionizing radiation [22]. Ionizing radiation decreases both RAD51 and DNA-protein kinase mRNA expression in human breast cancer cells. Treatment with melatonin before radiation caused a significantly higher decrease in RAD51 and DNA-protein kinase mRNA expression and sensitizes human breast cancer cells to ionizing radiation by reducing their proliferation, promoting cell-cycle arrest and decreasing the effectiveness of DNA repair [61]. Alonso-González et al. (2015) suggested that this modulatory action of melatonin on DNA repair could be mediated by p53, since melatonin increases p53 expression and this protein intervenes in the homologous recombination and in the non-homologous DNA end joining [61,62,63]. In human non-small-cell lung cancer and human colorectal adenocarcinoma cell lines, melatonin not only reduces the capability of tumor cells to repair DNA, but even enhances DNA damage exerted by irinotecan, a camptothecin analog used in clinic [64]. Besides its anti-oxidative actions by which this hormone protects DNA, melatonin also influences DNA repair by modulating some genes that induce protein synthesis related to DNA repair. 

The combination of melatonin with ionizing radiation decreased DNA repair efficiency and induced a decrease of the expression of RAD51 and BRCA1 in a colorectal carcinoma cell line. Melatonin increased sensitivity of colorectal cancer cells to ionizing radiation and its oncostatic actions were corroborated in *in vivo* xenograft tumor models in mice exposed to γ-ray radiation [65]. Moreover, pre-treatment with melatonin before 2–8 Gy ionizing radiation, increased the expression of Cdkn1 and RAD50 in rat peripheral blood and regulated DNA double strand breaks repair [66].

### 6.4. Modulation of Angiogenesis

Since the formation of new blood vessels plays an outstanding role in cancer growth, the inhibition of angiogenesis has been proposed as an adjuvant therapy in cancer treatment to maximize its effectiveness. In this sense, angiogenesis inhibitors enhance ionizing radiation effects in the treatment of non-small cell lung cancer [91]. Thus, at this moment, anti-VEGF/VEGFR (vascular endothelial growth factor/vascular endothelial growth factor receptor) monoclonal antibodies, antisense suppression of VEGF, VEGFR tyrosine kinase inhibitors, viral-directed targeting of VEGFR signaling or the use of endogenous angiogenic inhibitors are under investigation as potential adjuvants to radiation in the cancer treatment [92].

Melatonin exerts oncostatic actions in different cancer types through several mechanisms, among them the antiangiogenic actions [46,47,93]. Recently, González-González et al. (2019) described that melatonin is able to potentiate the usefulness of radiation by increasing antiangiogenic actions and by neutralizing pro-angiogenic actions induced by ionizing radiation, through modulating several steps of the angiogenic process [60]. They described that ionizing radiation inhibits cell proliferation, migration and tubular network formation. Interestingly, in co-cultures of human breast cancer and endothelial cells, treatment with melatonin has a synergic effect and potentiates the inhibition induced by radiation of some pro-angiogenic factors, like VEGF and ANG-2, on different steps of the angiogenesis. Besides, melatonin neutralizes the stimulatory effect of ionizing radiation on endothelial cell permeability and on the expression of some proangiogenic genes, like FGFR3, TGFα, IGF-1, KDR, JAG1, MMP14, CXCL6, CCL2, ERK1, ERK2 and AKT1. This melatonin potentiation of antiangiogenic effects induced by radiation was also described in an *in vivo* model of angiogenesis study, the chick chorioallantoic membrane assay, where melatonin enhanced the decrease of vascular area exerted by radiation. The authors described that these melatonin effects could be mediated through the potentiation of the inhibitory effect exerted by ionizing radiation on the activation of *p*-AKT (protein kinase B) and *p*-ERK (extracellular signal regulated kinase). In addition, the effectiveness of radiation can be reduced by some side effects and melatonin pre-treatment counteracts these negative actions of radiation [60].

### 6.5. Abolition of Inflammation

As a result of radiotherapy, short and long term side effects often appear and many of them are linked to the inflammatory response, especially at high doses of radiation. Immune cells infiltration and cytokine secretion play a principal role in tumor response to irradiation. After irradiation, different mechanisms of cell death are triggered and can stimulate different signaling pathways in immune cells with changes in the cytokines production in both irradiated and non-irradiated tissues. Some of the molecules implicated in radiation-induced immune response are cytokines, like IL-4, IL-5, IL-6, IL-10, IL-12, IL-18, IL-33, IL-1β and IFN-γ, transcription factors, like NF-кB, TNF-α, protein kinases, like MAPK, or growth factors like IGF-1, TGF-β, bFGF and PDGF. Melatonin is a modulator of the immune response to radiation. Immune cells, such as T cells, B cells, or macrophages, express melatonin receptors [91,94]. Melatonin treatment reduces DNA damage and ameliorates the effects of radiation on peripheral and bone marrow lymphocyte numbers [67]. In addition, melatonin stimulates the production of IL-2, IFN-γ, GM-CSF, IL-3, IL-4, IL-10 and IL-6, with an increase in activity of NK and production of macrophages, neutrophils, granulocytes and erythrocytes [67]. Melatonin behaves as an anti-inflammatory molecule and reduces the expression of TNF-α, IL-1β or IFN-γ, Th1 cytokines, which are involved in inflammation and on the contrary, it stimulates Th2 response. In lung cancer, melatonin treatment before radiation reduces the increase of TNF-α, IL-6 and TGF-β induced by ionizing radiation [68]. In many kinds of cancer, the increase of cytokines after radiation is mediated through NF-кB [95]. It is known that melatonin down-regulates the expression of NF-кB [67]. The lung is one of the most sensitive organs to the effects of radiation and side effects such as inflammatory response often turns into a main problem. The up-regulation of IL-4 signaling pathways activates the expression of duox1 and duox2, which mediate ROS production and stimulation of infiltration of macrophages, lymphocytes and mast cells. In rats irradiated with 15 Gy ^60^Co gamma rays, melatonin treatment before irradiation reversed all these changes and attenuated the inflammatory cells infiltration [69]. All these immunomodulatory actions of melatonin improved the survival of patients after radiotherapy.

Another main factor that mediates inflammatory response to radiation is the COX-2. The anti-oxidative and anti-inflammatory actions of melatonin included both inhibition of COX-2 and a general decline in the expression of transcriptional factors that mediate the inflammatory response to radiation [96]. In addition, in human pre-adipocytes exposed to ionizing radiation, melatonin inhibited COX-2 mRNA expression and this inhibition has been associated with local estrogen biosynthesis. The inhibition of cyclooxygenases expression in pre-adipocytes decreased the production of PGE_2_ and consequently, reduced the intracellular levels of cAMP and therefore the activation of aromatase promoter II. The final results are a decrease of aromatase expression and a reduction of estrogens production in pre-adipocytes [60].

### 6.6. Induction of Apoptosis

Apoptosis is the most common mechanism of cell death that is activated after irradiation of tumor cells and it is associated with a reduction of tumor development. Radiation targets DNA, either directly or through the generation of free radicals. When cells are not able to repair the damaged DNA they die through different mechanisms of cell death such as autophagy, mitotic catastrophe, senescence or apoptosis. The degree of apoptosis depends on some factors like the dose of radiation and the type of cells. Cells are going to be more sensitive to apoptosis in the face of stress conditions when they exhibit a high proliferation rate, high expression of pro-apoptotic genes and low expression of anti-apoptotic genes. Melatonin, contrary to what happens in normal cells where it has anti-apoptotic actions, potentiates apoptosis in malignant cells. In ovarian cancer cells, melatonin enhances the apoptosis induced by laser irradiation [54]. Melatonin sensitization of cancer cells to the induction of apoptosis after ionizing radiation is performed through several mechanisms, which have been summarized in a recent review [70]. In malignant hematological cells, melatonin treatment up-regulates Akt which induces ROS production and activate the Fas/FasL pathway, one of the main targets involved in induction of apoptosis in cancer cells after radiation and/or chemotherapy [70]. The levels of the tumor suppressor protein p53 are augmented in cells under genotoxic stress, like ionizing radiation, and induce apoptosis, cell-cycle arrest, senescence and DNA repair [58,97]. Conversely, the down-regulation of p53 has been associated with inhibition of both intrinsic and extrinsic pathway of apoptosis and tumor growth. In addition, the up-regulation of p53 potentiates the response of cancer cells to radiation via induction of apoptosis [98]. Melatonin through an increase of p53 mRNA expression sensitized human breast cancer cells to the effects of ionizing radiation [56]. Nooshinfar et al. (2016) described that melatonin potentiated the activation of p53 and p21 and the up-regulation of Bax expression induced by arsenic trioxide that lead to apoptosis [71]. Another way to regulate apoptosis is through the modulation of mitochondrial metabolism. The kind of metabolism determines the response to the pineal hormone. Melatonin is not able to modulate mitochondrial respiration in cancer cells with highly glycolytic profile [99]. In human breast cancer cells and in pituitary prolactin secretory tumor melatonin disrupted the activity of mitochondrial complexes inducing a mitochondrial dysfunction that leads to apoptosis. Melatonin also sensitizes cancer cells to radiation through regulation of NF-кB. NF-кB is a transcription factor involved in the induction of cell death, in the intrinsic pathway of apoptosis and in the generation of many inflammatory cytokines. Radiation increases NF-кB expression in cancer cells and melatonin inhibits, on some of them such as thyroid cancer cells, the phosphorylation and nuclear expression of NF-кB/p65, which induces apoptosis and a decrease of tumor invasive markers [55]. The activation of MAPKs (Mitogen Activated Protein Kinases) is also associated with proliferation and apoptosis. Thus, Jun N-terminal kinase (JNK), p38 and extracellular-signal-regulated kinase (ERK) intervene in the modulation of apoptosis by interacting with other pathways like AKT and PI3K. PI3K activation up-regulates Bcl-2 through NF-кB, leading to apoptosis in cancer cells. In addition, an increased AKT expression is observed in several kind of tumors, such as breast, colon, ovarian, pancreatic or colorectal malignancies [100,101,102,103]. In many of them, melatonin stimulates apoptosis through the inhibition of PI3K [72]. In esophageal squamous cell carcinoma melatonin activates pro-apoptotic caspases through the inhibition of AKT [70]. In addition, the inhibition of COX-2 has been related with stimulation of apoptosis mediated by stimulation of TRAIL (TNF-related apoptosis-induced ligand) and FasL (Fas ligand) receptors which are associated with cell death [73]. The inhibition of COX-2 by melatonin enhanced apoptosis in hepatocellular carcinoma [72]. 

### 6.7. Differentiation of Pre-Adipocytes

**Figure 3:**
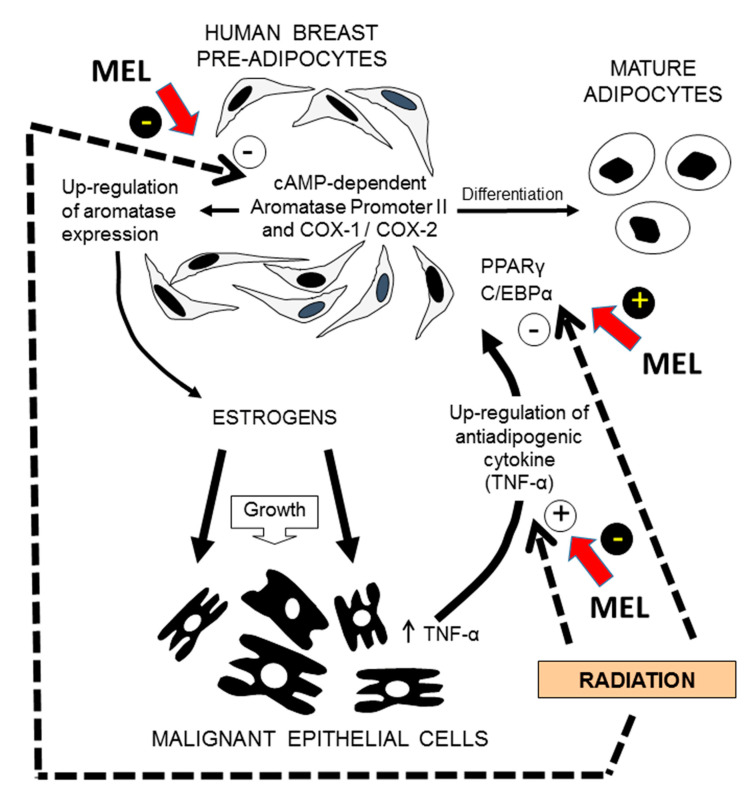
Modulation by melatonin of the pre-adipocytes response (differentiation, aromatase activity and expression) to ionizing radiation. In co-cultures of human breast pre-adipocytes and human breast cancer cells melatonin (MEL) counteracts the inhibitory action of radiation on differentiation of pre-adipocytes, by increasing C/EBPα (CCAAT/enhancer binding protein alpha) and PPARγ (peroxisome proliferator-activated receptor gamma) expression and by decreasing TNFα (Tumor necrosis factor α) expression, an anti-adipogenic cytokine secreted by breast malignant epithelial cells. Melatonin also potentiates the inhibition induced by radiation on aromatase activity and expression by increasing the down-regulation of aromatase promoter II, COX-1 and COX-2 expression. Figure drawn from data published by González-González et al. [87].

González-González et al. (2019) have recently showed that melatonin sensitizes breast fibroblasts to ionizing radiation. In co-cultures of human breast pre-adipocytes and human breast cancer cells, radiation is able to inhibit both proliferation and differentiation of breast fibroblasts through the down-regulation of the two main adipogenic transcription factors CCAAT/enhancer binding protein (C/EBPα) and peroxisome proliferator-activated receptor (PPARγ) and by stimulating the expression of TNFα (Tumor necrosis factor alpha), an anti-adipogenic cytokine secreted by breast malignant epithelial cells. In addition, radiation reduced aromatase activity and expression, another marker of undifferentiated pre-adipocyte phenotype, by decreasing aromatase promoter II and both COX-1 and COX-2 expression. Melatonin counteracted the inhibitory action of radiation on differentiation of pre-adipocytes, by increasing C/EBPα and PPARγ expression and by decreasing TNFα expression. Melatonin also potentiated the inhibition induced by radiation on aromatase activity and expression by increasing the down-regulation of promoter II, COX-1 and COX-2 expression (Figure 3). This study showed that, in a tumor microenvironment model, melatonin is able to modulate the response of pre-adipocytes (differentiation, aromatase activity and expression) to ionizing radiation [60].

### 6.8. Modulation of Metabolism

Cancer cells show an altered metabolic activity in comparison to normal cells and they obtain ATP mainly through glycolysis (which is enhanced) whereas mitochondrial oxidative phosphorylation activity is reduced, which is called Warburg effect [104]. Multiple regulators are involved in this metabolic switch: HIF-1α, NF-кB, Akt and mTOR signaling pathways, diverse oncogenes (c-myc), tumor suppressors, energy sensors, etc. [105]. Exposure to fractionated radiation with 0.5 Gy of X-rays of human cancer cells induces radio-resistance through an increase of aerobic glycolysis [106]. On the contrary, in lymphoma cells, the inhibition of glycolysis increased sensitivity of cancer cells to apoptosis [107]. In leiomyosarcoma melatonin inhibits aerobic glycolysis and this fact was related with an inhibition of metastasis [74]. The inhibition by melatonin of the Warburg effect in Ewing sarcoma has also been associated with an increase of cytotoxicity [75]. Blask et al. (2014) described, in tissue-isolated human breast cancer xenografts grown in nude rats that dim light at night (LAN)-induced melatonin suppression disrupts the circadian-regulated host/cancer balance among several important cancer inhibitory signaling mechanisms, inducing hyperglycemia and hyperinsulinemia in the host and enhancing aerobic glycolysis, lipid signaling and proliferative activity in the tumor [76]. The same group demonstrated that amplification of night-time melatonin levels in nude rats exposed to blue light during the day-time significantly reduces aerobic glycolysis and proliferative activity in human prostate cancer xenografts [77].

## 7. Clinical Trials with Melatonin as an Adjuvant to Radiotherapy 

Despite the amount of *in vivo* and *in vitro* studies about the efficacy of melatonin treatment associated to ionizing radiation, there are very few clinical trials that study its therapeutic usefulness in humans. One of the first attempts to associate melatonin and radiotherapy was made by Lissoni’s group, who studied the effects of melatonin treatment adjuvant to radiotherapy (60 Gy) in 30 patients with glioblastoma. Their preliminary study suggests that melatonin associated to radiation may ameliorate the quality of life and enhance the 1-year survival rate of patients [108]. 

However, in a randomized phase II clinical trial with patients with brain metastases that received radiation therapy in the afternoon (30 Gy in 10 fractions) and randomized to melatonin (20 mg), administered in the morning or in the evening, there were no significant benefits in the melatonin treated group in relation with the survival of the patients and their neurological deterioration [109].

Melatonin-containing emulsion treatment significantly decreased the appearance of dermatitis induced by radiation in stages 0-II breast cancer patients treated with breast whole radiation therapy (50 Gy) [110]. However, in other study, in a small group of cancer patients who underwent pelvic irradiation and were treated with or without melatonin, the appearance of another side effect of radiotherapy such as lymphocytopenia was studied. The main conclusion was that melatonin treatment did not modify the radiotherapy effects [111].

There is a meta-analysis of 21 clinical trials with patients with metastatic, solid tumor cancers. In some of them the combination of chemotherapy and melatonin decreased 1-year mortality and improved outcomes of complete response, partial response, and stable disease. In these studies, melatonin also significantly reduced asthenia, leucopenia, nausea and vomiting, hypotension and thrombocytopenia. The authors suggest that melatonin may benefit cancer patients who are also receiving chemotherapy, radiotherapy, supportive therapy, or palliative therapy by improving survival and ameliorating the side effects of chemotherapy. In this meta-analysis there is only one clinical trial with melatonin as an adjuvant to radiotherapy [112].

## 8. Conclusions

Improving the effectiveness of radiotherapy in cancer treatment can offer many benefits for cancer patients. The oncostatic properties of melatonin make it an interesting molecule in the treatment of cancer. The association of melatonin with radiotherapy has remarkable results both *in vitro* and *in vivo*. Radiosensitization of some types of cancer cells by melatonin have been related with an increase of reactive oxygen species and an enhancement of the cell-killing effect of radiation. In breast cancer cells melatonin treatment before ionizing radiation also increases the cytotoxic effects of radiation associated with the regulation of several proteins involved in estrogen biosynthesis. The up-regulation of p53, a regulator of effectors which promote cell-cycle arrest, DNA repair, apoptosis induction or a negative modulator of aromatase in the breast, has been suggested as the link between melatonin and its regulatory effect on breast cancer cells sensitivity to the ionizing radiation. Melatonin also reduces the capability of tumor cells to repair injured DNA and enhances DNA damage exerted by radiation by modulating proteins that intervene in both the homologous recombination and in the non-homologous DNA end joining. The usefulness of melatonin to potentiate the effects of radiation has been also related with an increase of anti-angiogenic actions and a neutralization of pro-angiogenic effects induced by ionizing radiation, through modulating several steps of the angiogenic process. Melatonin also behaves as an anti-inflammatory agent and reduces the expression some cytokines, which are involved in the inflammation induced by radiation. The sensitization of cancer cells by melatonin is also exerted by an increase of apoptosis induced by ionizing radiation. In tumor microenvironment, melatonin also may modulate the response of pre-adipocytes (differentiation, aromatase activity and expression) to ionizing radiation. The inhibition of glycolysis by melatonin in cancer cells also increases the sensitivity of cancer cells to the cytotoxicity of radiation. All these actions of melatonin make it a promising agent used as adjuvant to radiotherapy.

## Figures and Tables

**Table 1:** Mechanisms of melatonin radiosensitizing effects.

Radiosensitizing Effects of Melatonin		Cell Type/In *vivo* Model	Reference
**Oxidative Stress**	Increase ROS production	Head and neck squamous cell carcinoma	[52]
	Increase of *HSP70* expression	Ovarian carcinoma cells	[53]
	Activation of c-Jun NH2 kinase signaling	Human cervical cancer cells	[54]
	Inhibition of p65 phosphorylation	Thyroid cancer cells	[55]
**Modulation of estrogen biosynthesis**	Decrease aromatase, sulfatase and 17β-HSD1 activity	Breast cancer cells	[56]
	Increase sulfotransferase activity	Breast cancer cells	[56]
	Up-regulation of p53 protein	Breast cancer cells	[56,57,58]
	Inhibition of cell proliferation, migration and tubular network	Endothelial cells	[59]
	Decrease aromatase, sulfatase and 17β-HSD1 activity	Breast adipose fibroblasts	[60]
	Inhibition of COX enzymes	Breast adipose fibroblasts	[60]
**DNA repair mechanisms**	Decrease the effectiveness of DNA repair proteins	Breast cancer cells	[61]
	Up-regulation of p53 protein	Breast cancer cells	[61,62,63]
	Enhance DNA damage and reduce DNA repair mechanisms	Non-small-cell lung cancer cells Colorectal adenocarcinoma cells	[64]
	Increase sensitivity to ionizing radiation	Colorectal carcinoma xenografts tumor model	[65]
	Increase Cdkn1 and RAD50 proteins regulating DSB repair	Rat peripheral blood	[66]
**Modulation of angiogenesis**	Inhibition of pro-angiogenic factors (*VEGF, ANG2*)	Endothelial cells	[59]
	Decrease vascular area	Chick chorioallantoic membrane assay	[59]
	Inhibition of the activation of p-AKT and p-ERK	Endothelial cells	[59]
**Modulation of inflammatory response**	Reduce DNA damage	Peripheral and Bone marrow lymphocytes	[67]
	Reduce the expression of inflammatory cytokines	Lung cancer cells	[68]
	Reduce the expression of NF-kB, decreasing cytokines production	Lung cancer cells	[68,69]
	Decrease IL-4 signaling pathways, reducing ROS production and inflammatory cells infiltration	Rats irradiated with 15Gy ^60^Co gamma rays	[69]
**Induction of apoptosis**	Enhance the apoptosis induced by laser irradiation	Ovarian cancer cells	[54]
	Upregulation of Akt and activation of Fas/FasL pathway	Malignant hematological cells	[70]
	Upregulation of p53, p21 and Bax expression	Breast cancer cells	[71]
	Inhibition of AkT and activation of pro-apoptotic caspases	Esophageal squamous cell carcinoma	[70]
	Inhibition of COX-2 and stimulation of cell death receptors signaling pathways	Hepatocellular carcinoma	[72,73]
**Modulation of metabolism**	Inhibition of aerobic glycolysis and inhibition of metastasis	Leiomyosarcoma	[74]
	Inhibition of the Warburg effect	Ewing Sarcoma	[75]
	Inhibition of aerobic glycolysis, lipid signaling and proliferative activity	Tissue-isolated breast cancer xenografts rats	[76]
	Inhibition of aerobic glycolysis and proliferative activity	Tissue-isolated prostate cancer xenografts rats	[77]

## Abbreviations

ROS	Reactive Oxigen Species
HSP70	Hear Shock Protein 70
p65	Nuclear factor NF-kappa-B p65 subunit
17β-HSD1	17β-Hidroxysteroid-dehydrogenase type 1
CDKN1	cyclin-dependent kinase inhibitor 1
RAD50	Double Strand Break Repair Protein
DSB repair	Double Strand Break repair
COX-2	Ciclooxygenase-2
VEGF	Vascular endothelial growth factor
ANG-2	Angiopoietin-2
p-AKT	protein kinase B
p-ERK	extracellular signal regulated kinase
pNF-κB	Nuclear factor kappa-light-chain-enhancer of activated B cells
IL-4	Interleukine-4
p21	cyclin-dependent kinase inhibitor 1
Bax	BCL2 Associated X, Apoptosis Regulator
